# POSTER SESSION 1: Abstracts 105–384

**DOI:** 10.1002/pmf2.12023

**Published:** 2025-01-05

THURSDAY

January 30, 2025

10:30 AM – 12:30 PM

